# RacA-Mediated ROS Signaling Is Required for Polarized Cell Differentiation in Conidiogenesis of *Aspergillus fumigatus*

**DOI:** 10.1371/journal.pone.0149548

**Published:** 2016-02-18

**Authors:** Myoung-Hwan Chi, Kelly D. Craven

**Affiliations:** Plant Biology Division, Samuel Roberts Noble Foundation, 2510 Sam Noble Parkway, Oklahoma, United States of America; The University of Wisconsin—Madison, UNITED STATES

## Abstract

Conidiophore development of fungi belonging to the genus *Aspergillus* involves dynamic changes in cellular polarity and morphogenesis. Synchronized differentiation of phialides from the subtending conidiophore vesicle is a good example of the transition from isotropic to multi-directional polarized growth. Here we report a small GTPase, RacA, which is essential for reactive oxygen species (ROS) production in the vesicle as well as differentiation of phialides in *Aspergillus fumigatus*. We found that wild type *A*. *fumigatus* accumulates ROS in these conidiophore vesicles and that null mutants of *racA* did not, resulting in the termination of conidiophore development in this early vesicle stage. Further, we found that stress conditions resulting in atypical ROS accumulation coincide with partial recovery of phialide emergence but not subsequent apical dominance of the phialides in the *racA* null mutant, suggesting alternative means of ROS generation for the former process that are lacking in the latter. Elongation of phialides was also suppressed by inhibition of NADPH-oxidase activity. Our findings provide not only insights into role of ROS in fungal cell polarity and morphogenesis but also an improved model for the developmental regulatory pathway of conidiogenesis in *A*. *fumigatus*.

## Introduction

Fungi are not a homogenous collection of cells, but differentiate these cells into various shapes and sizes. In filamentous fungi, two morphological processes predominate, isotropic expansion and polarized growth. This is exemplified by the filamentous yeast, *Candida albicans*, which cycles between isotropic expansion and polarized growth [1]. In this case, there is a clear cut distinction between the isotropic yeast growth habit and the filamentous habit, and the switch between them appears to be activated following specific environmental cues, including nutritional starvation. In the case of the “true” filamentous fungi, the distinction between these growth habits is less clear cut, and the two forms are often mixed into the development of more complex and multicellular structures. Asexual development in *Aspergillus* species has been identified as a model of this dynamic morphogenesis because the asexual conidiophore is made up of a complex combination of isotropic and polarized cell growth. Aerial hyphae arise from foot cells to yield the conidiophore stalk, a structure characterized by unidirectional, polarized growth without branching, before switching to isotropic expansion to form a swollen vesicle [2]. This is followed by the emergence of rod-shaped sterigmata (metulae and/or phialides) on the surface of these vesicles. Finally, chains of asexual spores are produced from the phialides to complete the asexual conidiogenesis process. The development of sterigmata from the conidiophore vesicle is particularly fascinating because multiple polarized apexes are simultaneously differentiated from the single subtending cell (vesicle). It was also shown that septins, cellular markers of polarized growth, are localized to vesicle-phialide (or metulae) interface [3–5]. Despite numerous studies describing the *Aspergillus* conidiogenesis process in general, very little is known about the mechanisms underlying the complex projection and apical elongation of phialides or the stimuli that initiate this multi-directed, polarized growth habit.

Reactive oxygen species (ROS) produced by the NADPH oxidase complex, including the small GTPase Rac, have been implicated in the signaling and accurate functioning of several types of morphological development in filamentous fungi, including apical growth [6–11]. Previously, we reported an *Aspergillus fumigatus* strain deleted for *racA* (***Δ****racA*), and showed it to be required for the production of a plasma membrane-localized ROS signal necessary for apical growth [9]. As the ***Δ****racA* strain was defective in maintaining apical dominance and conidia production, we hypothesized that the apical projection of phialides from vesicles may also require a ROS signal. To verify this hypothesis, we employed an oxygen-enriched gel environment, in which the development of the conidiophore terminates just after the formation of phialides. This enabled clear microscopic observations to be made without pigmented conidia to obscure the process [12]. Here, we report the detailed phenotypic progression of conidiogenesis in the ***Δ****racA* strain within an oxygen-enriched gel environment and uncover a putative role for ROS signaling that appears to regulate conidiophore morphogenesis independently of, but simultaneously with, the central transcription regulatory cascade in *A*. *fumigatus*.

## Materials and Methods

### Fungal strains and growth conditions

*A*. *fumigatus* (Fresenius, 1863) strain AF293 [13] served as the wild type strain in this study. The *racA* null mutant (*ΔracA* strain; Δ*racA*::*Aspergillus parasiticus pyrG pyrG1*) was generated in our previous study [9]. Since the *racA* complement strain exhibited a normal phenotype with respect to conidiation [9], genetically demonstrating that the conidiation defects in the *ΔracA* strain were due to deletion of the *racA* gene, we didn’t include the complement strain in this study. All fungal strains were maintained on glucose minimal media (GMM, 1% glucose) plates including 1.5% (w/v) agar as previous described [14, 15]. Fungal colonies usually were maintained in the dark at 30°C, but were also grown at 25°C or 37°C for temperature shifting test(s). Osmotic stress was created by amending GMM with a sorbitol solution (Sigma, S-1876) to a final concentration of 1 M. Carbon starvation stress was induced by subtracting glucose from GMM. Plates were placed in the incubator upside down to prevent condensation on the lid and accumulation of carbon dioxide in the culture, which may affect conidiogenesis.

### Observation of conidiophore development

Microscopic observation of conidiogenesis in the media-air interface was performed as described previously [12]. For ROS staining, thin sections of agar blocks were incubated in 0.5mM NBT (IscBioExpress, 0329-1G) solution at room temperature for 30 minutes. Cell wall staining of aerial conidiophores was done by embedding a thin section of the agar block in 0.7% agarose gel and incubating in 25μM Calcofluor White (CW, Sigma, F3543) solution for 1h. Microscopy was done using Olympus BX41 and SZX12 microscopes, Olympus DP71 CCD camera (Olympus America Inc.), and Leica TCS SP2 AOBS confocal system (Leica Microsystems, Wetzlar, Germany). All observations described in this study were repeated more than three times to confirm reproducibility.

### Inhibition of conidiophore development by DPI treatment

Colonies of AF293 were grown under cellophane membrane and covered with glass coverslips to prevent unintended conidiophore development (GMM, 30°C, dark condition). Four-day-old colonies were exposed to high-oxygen for 18 hours and subsequently to fluorescent light for 6 hours to synchronize induction of conidiogenesis. Colonies were sliced into thin-sections of agar block (5 mm × 3 mm × 1~2 mm) as described previously [12]. Agar blocks containing mature mycelium (neither colony periphery nor center) were selected to minimize age variation and treated with DPI. Sliced agar blocks were placed in 8-well culture slides (Fisher Scientific, 08-774-26), and submerged with 400 μl of liquid GMM containing 0, 1, and 2 μM of DPI (Diphenyleneiodonium chloride, Sigma, D2926) respectively. Since the stock solution of 10 mM DPI was dissolved in DMSO, 100 ppm of DMSO was added in a 0 μM control treatment. The culture slides containing agar blocks were incubated in high-oxygen conditions under fluorescent light for 16 hours (room temperature). The agar blocks were then fixed with FAA (Formalin–acetic acid–alcohol) for 30 min, washed twice with 1x PBS, and mounted to slides for microscopy. The numbers of conidiophores that were observed in each stage of development, from vesicle to mature phialides, were counted. The experiment was repeated three times, and the average for each stage was transformed into a percentage or ratio each accounted for of the total. Ratios were compared between 0, and 1 uM DPI. No conidiophores were observed in the 2 uM DPI condition. For comparison, conidiogenesis of the ***Δ****racA* strain was also induced as described above, but the colony of ***Δ****racA* strain was not submerged in GMM.

### Isolation of RNA and quantitative reverse transcriptase PCR analysis

Mycelia were grown on the surface of cellophane membrane placed on GMM agar media for 4 days at 30°C and harvested by peeling the cellophane membrane off from the media surface. RNA extraction and quantitative PCR were performed as described previously [12].

## Results

### A reactive oxygen burst occurs in vesicles of *A*. *fumigatus* prior to phialide formation

**Fig 1:**
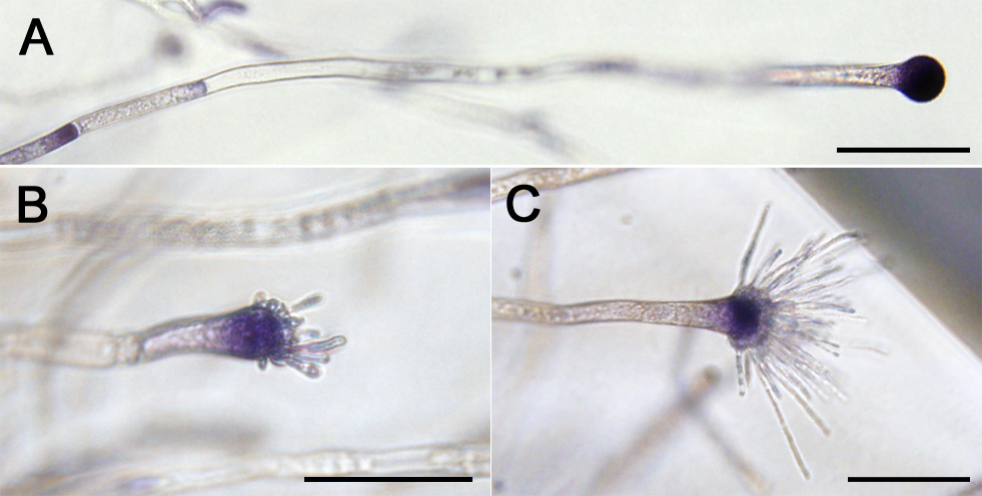
ROS accumulates in conidiophore vesicles of *A*. *fumigatus*. Agar blocks including the embedded conidiophores were prepared from an *A*. *fumigatus* colony grown on GMM agar plate (30°C, 4 days after inoculation) by a described sectioning method (Chi and Craven, 2013), and stained with 0.5 mM nitroblue tetrazolium (NBT). Pictures represent various developmental stages of immature conidiophores; **(A)** vesicle stage, **(B)** after phialide budding, **(C)** and phialide elongation stage. Bars = 100 μm.

To investigate the relationship between ROS production and the conversion between isotropic and apical growth in conidiophore development, *A*. *fumigatus* wild-type strain AF293 was observed in an oxygen-enriched gel environment, a condition we have previously shown to stimulate this transition [12]. We visualized ROS localization using nitroblue tetrazolium (NBT) staining [16], and found high levels of ROS had specifically accumulated in the conidiophore vesicles (Fig 1). These levels were prevalent in vesicles at all stages of development including during the subsequent origination of young phialides (Fig 1B and 1C). Little or no ROS was detected in the phialides as they emerged from the vesicles (Fig 1B) or during their elongation and maturation (Fig 1C). This might indicate that the ROS is involved in early phialide differentiation from the subtending vesicle.

### Deletion of the small GTPase RacA causes defects in vesicular ROS accumulation and phialide development

**Fig 2:**
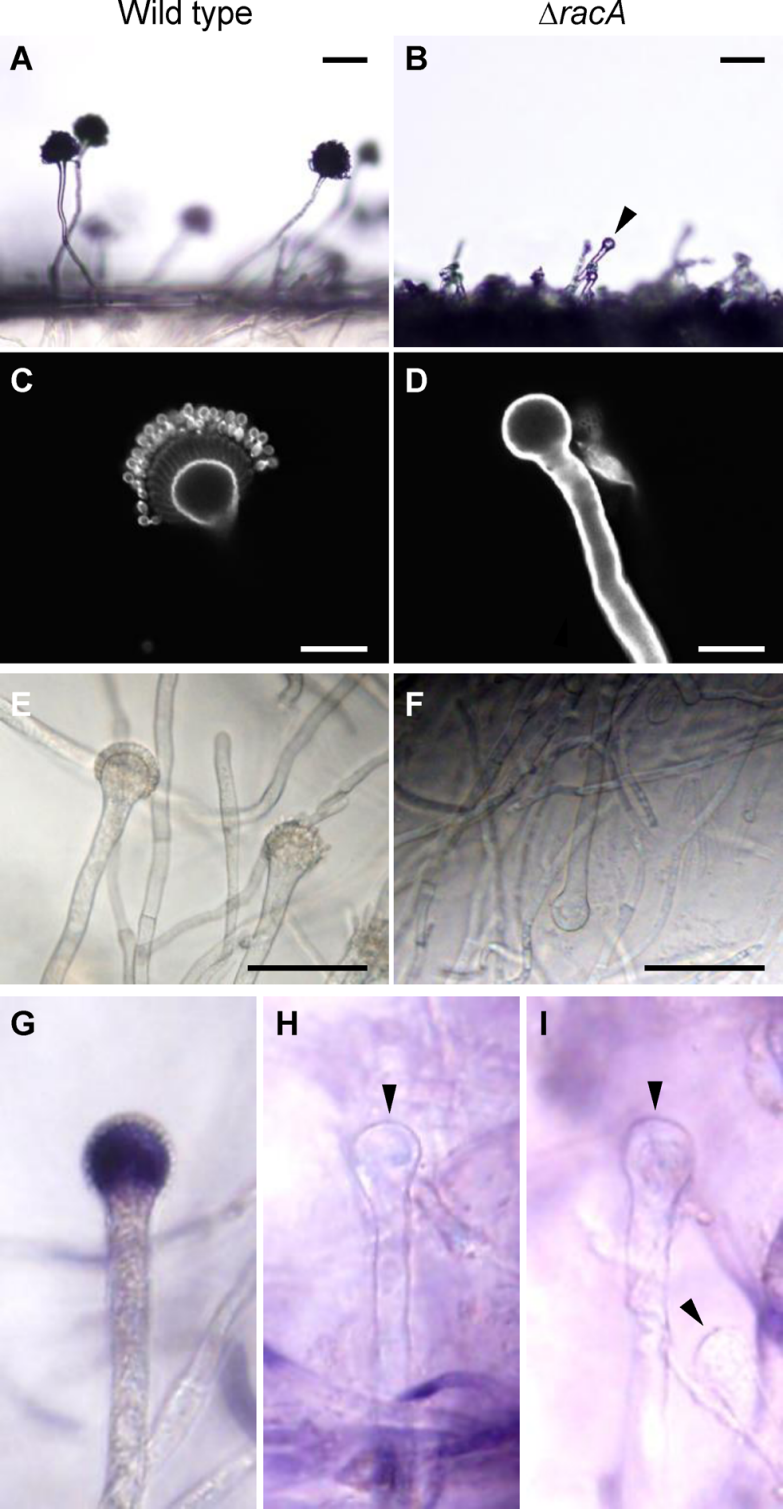
The *A*. *fumigatus ΔracA* is defective in phialide development. Agar blocks including aerial or embedded conidiophores were prepared from an *A*. *fumigatus* colony grown on GMM agar plate (30°C, 4 days after inoculation). Vesicles of the *ΔracA* are indicated with black arrowheads. **(A and B)** Aerial conidiophores of the wild type (A) and the *ΔracA* mutant (B). Bars = 50 μm. **(C and D)** Conidiophores of the wild type (C) and the *ΔracA* mutant (D) stained with 25 μM Calcofluor White. Bars = 15 μm. **(E and F)** Agar-embedded conidiophores of the wild type (E) and the *ΔracA* mutant (F). Bars = 50 μm. **(G—I)** Embedded conidiopohres of the wild type (G) and the *ΔracA* mutant (H and I) stained with 0.5 mM NBT.

Our previous data suggested that the small GTPase, RacA, is required for maintenance of apical dominance in expanding hyphae of *A*. *fumigatus* [9]. Furthermore, the ***Δ****racA* mutant was defective in conidiophore development, with branched and irregularly shaped conidiophore stalks [9]. Since ROS were highly accumulated in conidiophore vesicles (Fig 1), we hypothesized that RacA is involved in this localized burst of ROS as well as the subsequent transition from isotropic (vesicles) to apical growth (phialides). To evaluate this hypothesis, we investigated conidiophore development of the ***Δ****racA* mutant in detail. This development was observed in both a normal aerial environment (Fig 2A–2D) as well as an oxygen-enriched gel environment for clear vesicle imaging and ROS staining (Fig 2E–2I). Most obviously, the vesicles of the ***Δ****racA* mutant failed to produce phialides. Conidiophores of the ***Δ****racA* mutant exhibited a “barren” phenotype that was arrested in the isotropic stage (Fig 2D), whereas conidiophores of the wild-type stain developed many phialides from the vesicle (Fig 2C). Furthermore, the “barren” phenotype of the ***Δ****racA* mutant coincides with a lack of ROS accumulation in the subtending vesicles (Fig 2G–2I). These observations suggest that RacA is required for ROS accumulation in the vesicle and the ultimate development of phialides.

### Various environmental stresses partially restore conidiation in the *ΔracA* mutant

**Fig 3:**
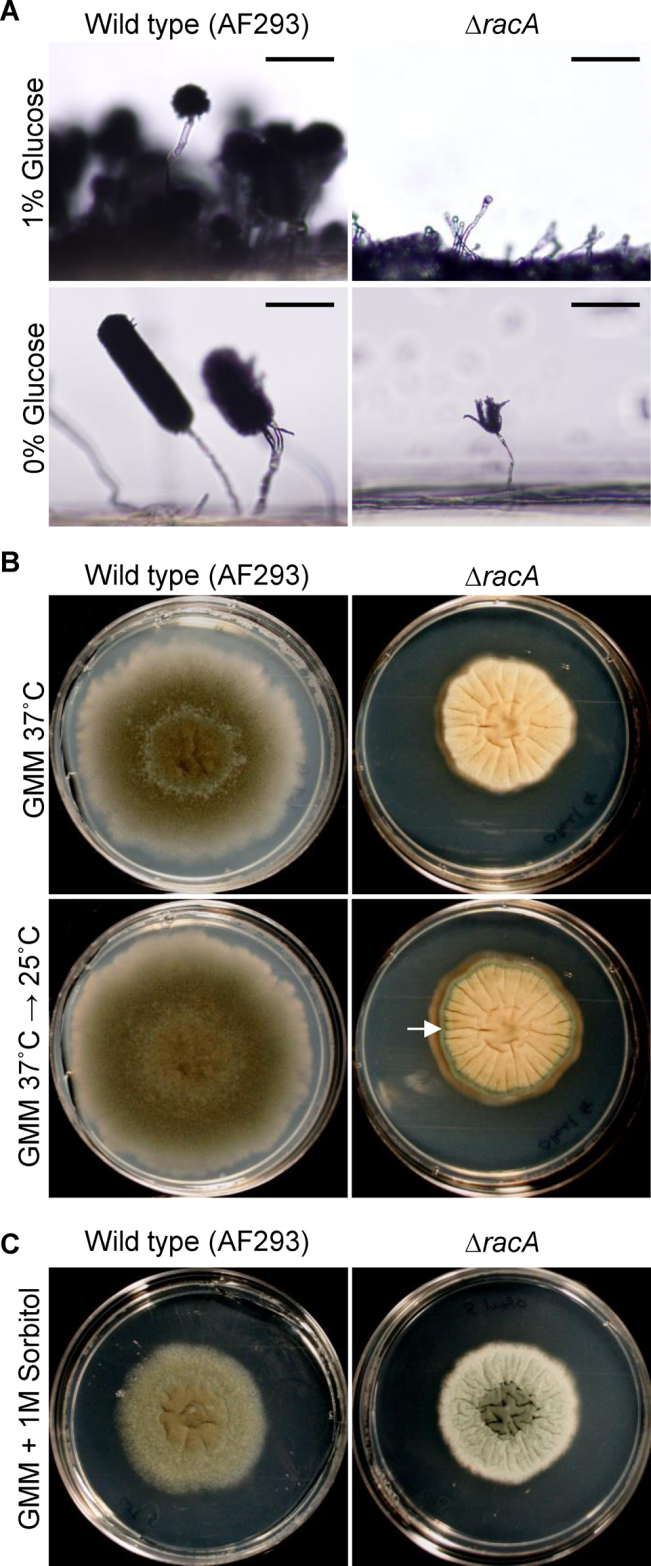
Conidiation in the *ΔracA* mutant is partially restored in some stress conditions. **(A)** Aerial conidiophores of the wild type and the *ΔracA* mutant on minimal media with (top) or without (bottom) 1% glucose. Colonies were grown for 6 days at 37°C. Bars = 100 μm. **(B)** Colonies of the wild type and the *ΔracA* mutant on GMM grown at 370°C (top) or initially at 37°C, then switched to 25°C for 24 hours (bottom). White arrow indicates zone of conidiation. **(C)** The wild type and the ***Δ****racA* mutant on GMM with 1 M sorbitol 6 days at 37°C.

We previously found that the conidiation defects we observed in the ***Δ****racA* mutant could be at least partially recovered in nutrient rich Sabouraud dextrose media [9], suggesting that some environmental factors may complement the developmental defect in the ***Δ****racA* mutant. To follow up, we tested several additional environmental conditions including carbon starvation, temperature shifting, osmotic stress (1M sorbitol), and additional nutrition (1% yeast extract). We observed at least partial recovery of phialide formation and conidia development in carbon starvation (Fig 3A, bottom right panel), temperature shifting from 37°C to 25°C (Fig 3B, bottom right panel), and 1M sorbitol conditions (Fig 3C, right panel). No recovery or phenotype change of the ***Δ****racA* mutant was observed in the 1% yeast extract condition. Intriguingly, the recovery pattern varied depending upon the condition tested. In carbon starvation conditions, the wild-type produced a reduced number of stalks with extremely long conidial chains (Fig 3A, bottom left panel). In this condition, the ***Δ****racA* mutant recovered some conidia production, but the length of conidial chains as well as the number of conidiophore stalks were still greatly reduced compared to the wild type (Fig 3A, bottom right). To test the effect of temperature shifting, colonies of the wild type and the *ΔracA* mutant on GMM grown initially at 37°C, then switched to 25°C for 24 hours. In the temperature shifting condition, while the wild-type strain didn’t exhibit a significant difference before and after the shift, the ***Δ****racA* mutant produced mature conidiophores and conidia (a green ring in Fig 3B, bottom right) in the colony periphery. The color of the conidiation region in the ***Δ****racA* mutant was much lighter than the wild-type. While the mechanism behind this phenotype is unclear, it is interesting to note that the conidia production coincides with the youngest, actively growing region of the fungal mycelium. Also of interest, no recovery was observed when the ***Δ****racA* mutant was shifted from 25°C to 37°C (data not shown). Yet a third recovery phenotype was observed when the ***Δ****racA* mutant was placed in an osmotically stressful condition (Fig 3C). In contrast to the peripheral recovery noted in the temperature shifting condition, conidiation recovery of the ***Δ****racA* mutant in 1M sorbitol was mostly restricted to the colony center (Fig 3C, right panel). Furthermore, osmotic stress differentially affected the overall growth rate of the wild-type strain and the ***Δ****racA* mutant, with an obvious growth retardation of the former but no significant growth reduction of the ***Δ****racA* mutant (compare growth patterns in Fig 3B and 3C). These findings suggest the presence of some secondary signal, seemingly independent of RacA and induced upon exposure to certain stress conditions, that temporarily and partially complements the conidiation defects of the ***Δ****racA* mutant.

### Recovery of phialide development in the *ΔracA* mutant coincides with atypical accumulation of ROS in the conidiophores

**Fig 4:**
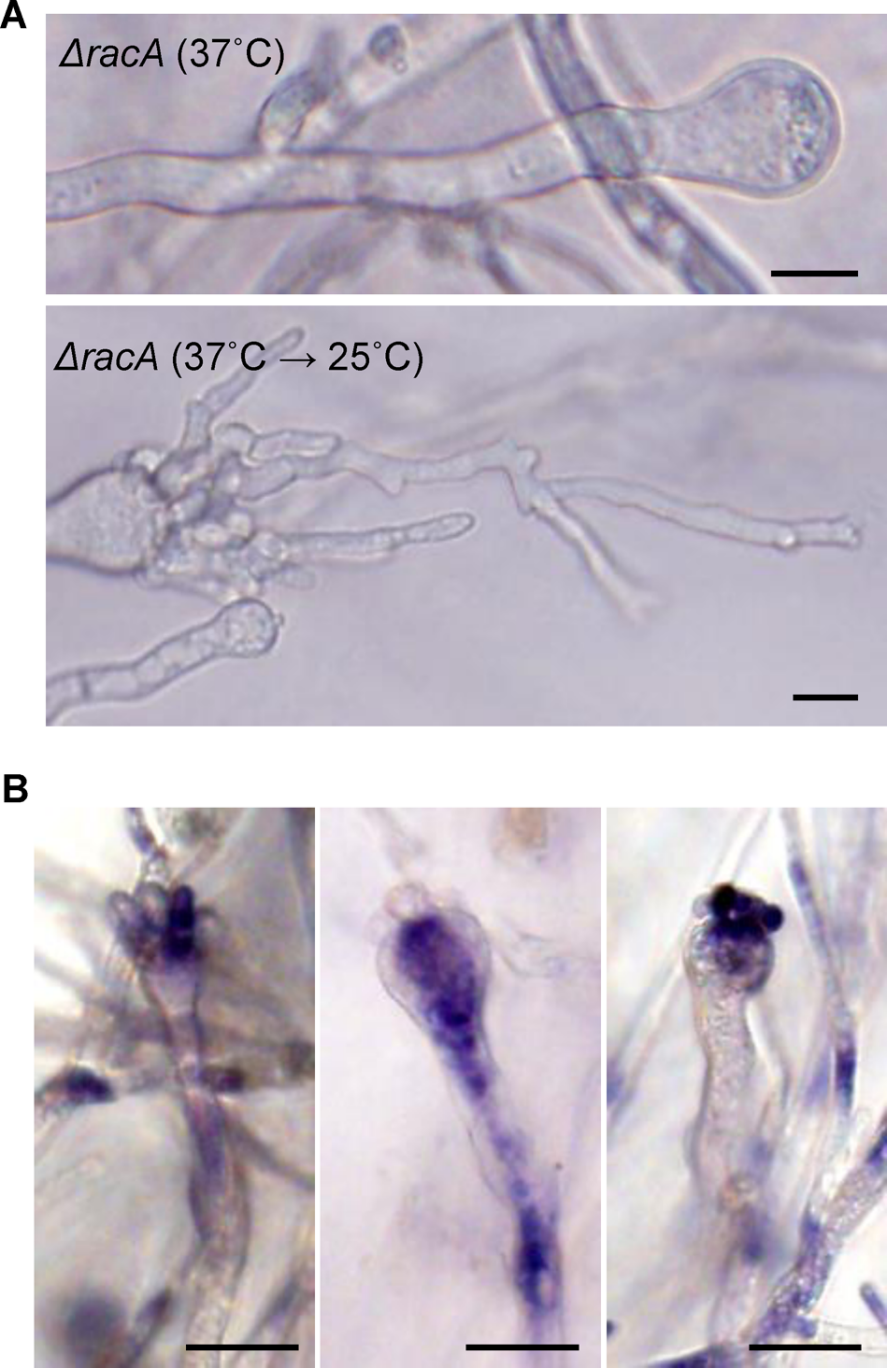
Phialide development and ROS production in the *ΔracA* mutant were partially restored at temperature shifting condition. **(A)** Conidiophore development of the *racA* mutant grown at 37°C only (top) or initially at 37°C, then switched to 25°C (bottom). Bars = 10 μm. **(B)** ROS distributions in the *ΔracA* mutant conidiophores after temperature switching from 37°C to 25°C. Bars = 20 μm.

Since the “barren” phenotype of the ***Δ****racA* mutant coincides with failure of ROS accumulation in the vesicles (Fig 2G–2I), we hypothesized that ROS is required for phialide emergence from the vesicle(s) of *A*. *fumigatus*. In support of this notion, we observed ROS accumulation in the vesicles subtending recovered phialides of the ***Δ****racA* mutant in the temperature shift (37°C to 25°C) condition (Fig 4A). Interestingly, these recovered phialides of the ***Δ****racA* mutant lacked apparent apical dominance and instead exhibited an unusual, branched pattern (Fig 4A). In addition, the ROS accumulated in unusual patterns, even in emerging phialides and in the middle of stalks (Fig 4B). Along with the light pigmentation in the aerial conidiophore, this suggests that the temperature shift (37°C to 25°C) can induce ROS, phialide emergence and conidia production from the ***Δ****racA* conidiophore, but it is still not sufficient for maintaining ROS distribution, apical dominance of the phialides, and pigmentation in conidia.

### Inhibition of NADPH oxidase activity limited phialide elongation

**Fig 5:**
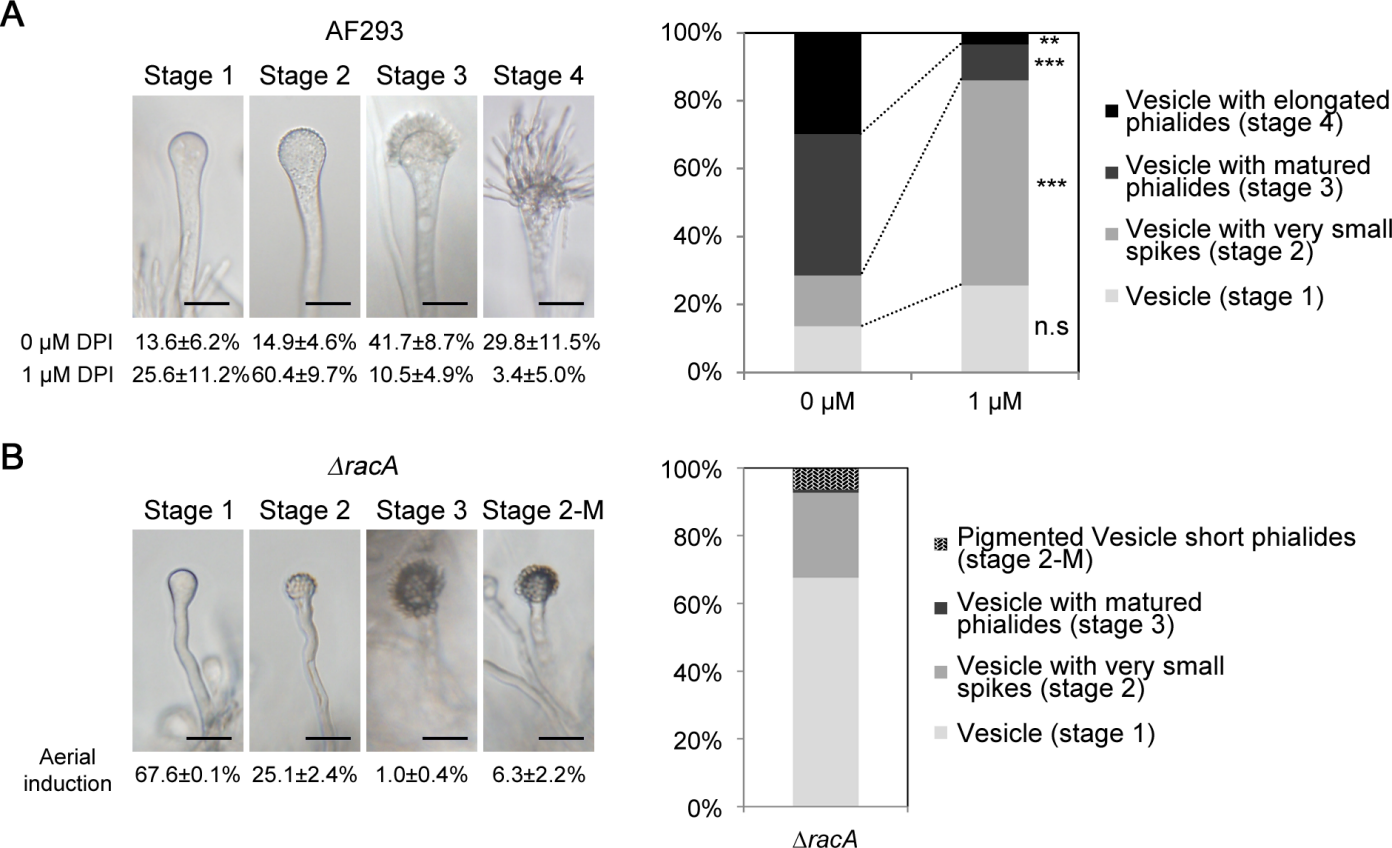
Treatment of NADPH-oxidase inhibitor partially mimics the *ΔracA* mutant phenotype. **(A)** Four representative developmental stages of AF293 phialides are shown in the top panel (see Results for detailed description). Bars = 20 μm. After 16 hour treatment in liquid GMM (with or without DPI), the number of conidiophores grouping into each of four defined categories were counted and the ratio of each to the total was calculated (for 0 μM condition, n = 135, 133, and 179; for 1 μM condition, n = 78, 65, and 94, respectively). Means ± s.d. from three biological replicates are displayed under each category. A visualized graph of the ratio is shown in the right panel. Note that ratios determined for 1 μM treatment are significantly different from untreated, as evaluated by t-test (n.s = not significant, **; p<0.05; ***; p<0.01). **(B)** Four representative developmental stages of the ***Δ****racA* phialides are shown in the left panel [note that there is stage 2-M (mature) instead of stage 4 because of aerial induction]. Bars = 20 μm. After 22 hours of air and light exposure, the number of conidiophores falling in each category were counted and calculated in ratio (n = 77, and 127, respectively. Means ± s.d. from two biological replicates were displayed for each category. Visualized graph of the ratio is shown in the right panel.

ROS accumulation was affected in the vesicles of the ***Δ****racA* mutant, which typically just precedes phialide development. Further, it is well known that the NADPH-oxidase (NOX) enzyme is responsible for ROS production, and it’s localization to the plasma membrane is guided by the Rac GTPAse [7]. Thus, we hypothesized that NADPH oxidase activity is required for phialide development in *A*. *fumigatus*. To evaluate this hypothesis, we investigated wild type conidiophore development following treatment with DPI, a known NADPH-oxidase inhibitor [17]. This detailed observation lead us to categorize conidiophore development into 4 distinct stages; conidiophores with smooth vesicles (stage 1), vesicles with very short “spikes” (initial phialides, stage 2), vesicles with mature (normal) phialides (stage 3), and vesicles with elongated phialides (stage 4) (Fig 5A). To synchronize the induction of conidiogenesis, 4-day-old colonies grown in anaerobic, embedded, and dark conditions were exposed to air and light (see Materials and Methods), and then thin-sectioned agar blocks containing emerging aerial hyphae were submerged in DPI solution (or 0 μM DPI control) and monitored for conidiophore development. In 0 μM DPI, over 70% of vesicles had developed to become fully mature (stage 3) or had elongated phialides (stage 4). In contrast, the addition of 1 μM DPI resulted in the arrest of a majority of conidiophores (~60%) in stage 2 (Fig 5A). Further, no or very few conidiophores were observed when the DPI concentration was 2 uM or higher (data not shown). This provides evidence that NADPH-oxidase activity is required for phialide elongation. The ***Δ****racA* strain produced no or very few conidiophores in the submerged condition regardless of DPI concentration (data not shown). Therefore, we could only compare the DPI-treated wild type strain with the ***Δ****racA* strain in a more normal aerial condition. The major portion (68%) of ***Δ****racA* vesicles was exhibiting the same “barren” phenotype (stage 1). Intriguingly, an additional portion (25%) of the ***Δ****racA* vesicles were arrested in stage 2 (Fig 5B), which together with stage 1 and 2 accounts for greater than 90% of all conidiophores examined. We further found that about 6% of the ***Δ****racA* vesicles were highly pigmented without having normal sized phialides (Fig 5B, Stage 2-M). Indeed, very few (1%) of the ***Δ****racA* vesicles developed normally-elongated phialides (Fig 5B, Stage 3). This might indicate that RacA function is required for both transitions from stage 1 to 2 as well as stage 2 to 3, while NADPH oxidase activity seems to be confined to the latter.

### Expression of central regulatory genes in the *ΔracA* mutant

**Fig 6:**
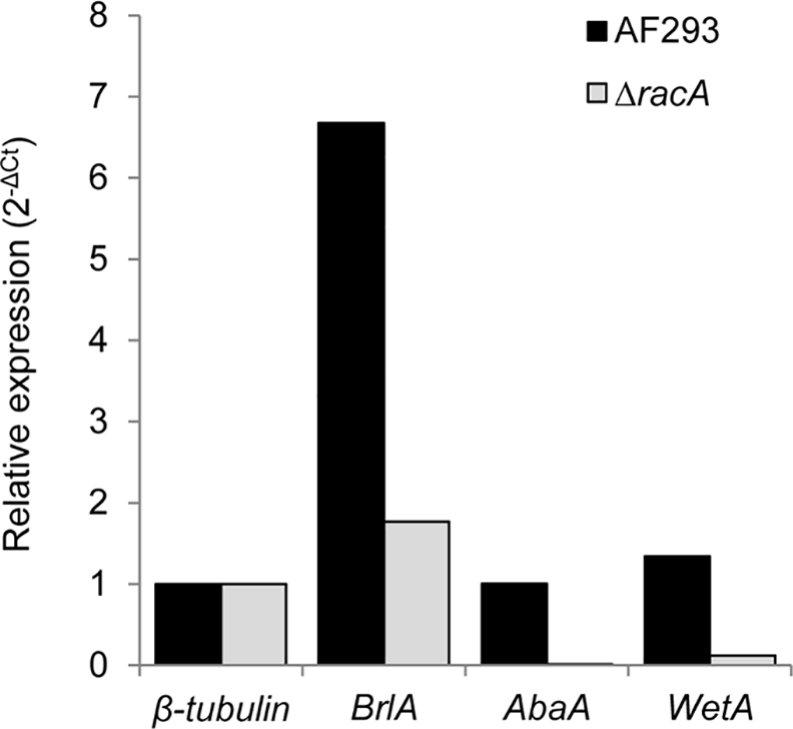
Expression of central regulatory genes are reduced in the *ΔracA* mutant. Total RNA extracted from AF293 and the *ΔracA* colonies grown on GMM agar media from on top of a cellophane membrane for 96 hours at 30°C. Bars are mean values from three technical replicates normalized with Ct values of β-tubulin gene.

To further elucidate the genetic mechanism(s) involved in the defective conidiogenesis of the ***Δ****racA* mutant, we examined the expression level of central regulatory genes controlling conidiogenesis (*AfubrlA*, *AfuabaA*, and *AfuwetA*) [18, 19]. The expression of *AfubrlA*, *AfuabaA*, and *AfuwetA* in the ***Δ****racA* mutant was about 27%, 1% and 9% of the wild type strain, respectively (Fig 6). These numbers correspond well with the defective phenotypes of the ***Δ****racA* mutant; reduced conidiophores (*AfubrlA*), vesicles with no or very short phialides (*AfuabaA*), and light pigmentation of conidia (*AfuwetA*).

## Discussion

### Oxidative signaling in conidiogenesis

Reactive oxygen species (ROS) generated from the NADPH-oxidase (Nox) complex, itself recruited to the plasma membrane by the Rac GTPase, act as critical signals for developmental differentiation of filamentous fungi [20–23]. We reported previously that conidia production is severely impaired in the *racA* mutant of *A*. *fumigatus* [9]. Here, we report further that this defect appears to be due to the requirement of the RacA-mediated ROS signal in phialide elongation. We have shown that treatment with the NADPH-oxidase inhibitor (DPI) stalls phialide development in the initial stage (Fig 5A, stage 2). DPI treatment doesn’t seem to block the early emergence of phialides because the increased ratio of stage 1 vesicles in DPI-treated samples from DPI-free samples was not significant (Fig 5A, stage 1). This suggests that the ROS signal generated by NADPH-oxidase may be important in phialide elongation rather than initiation from the vesicle. However, since the ***Δ****racA* mutant didn’t produce *any* conidiophores in the submerged condition we tested (similar to the wild-type strain treated with high concentrations of DPI), we cannot exclude the possibility that this partial suppression of phialide development may be due to incomplete suppression of NADPH oxidase activity. Further studies using additional Nox gene mutants should clarify this. Importantly, the defect in phialide budding observed in the ***Δ****racA* mutant could be compensated by various environmental factors that seemed to be unfavorable for vegetative growth, including low temperature, osmotic stress, and carbon starvation (Fig 3). The defect in ROS accumulation in conidiophore vesicles in the ***Δ****racA* mutant was also recovered by the temperature shift (Fig 4). This indicates that ROS produced in both a RacA-dependent or independent manner may serve as the intracellular signal(s) for phialide elongation, although the latter doesn’t seem to be sufficient for maintaining apical dominance of the phialides (Fig 4A) and the source of this ROS remains unknown (possibly mitochondria or alternative oxidases).

The small GTPase Rac is known to be required for cellular morphogenesis and controlling cell polarity in many fungi. In several dimorphic fungi including *Cryptococcus neoformans*, *Candida albicans*, and *Yarrowia lipolytica*, it has been shown that Rac is required for the dimorphic transition from yeast form to filamentous form [24–26]. In *Neurospora crassa*, RAC-1 is required for formation of conidial anastomosis tubes (CATs) whereas CDC-42, the other Rho type GTPase, is required for germ tube formation. Appressoria of the rice blast fungus, *Magnaporthe oryzae*, undergo a very similar differentiation process that involves a transition from isotropic (appressorium) to polarized (penetration peg) cell growth [27]. All of these transitions require ROS synthesis by NADPH oxidases [20, 28], which directly interact with Rac [7]. We found additional evidence that this RacA-mediated ROS signal is also involved in the elongation of phialides from their subtending vesicles in *A*. *fumigatus*.

**Fig 7:**
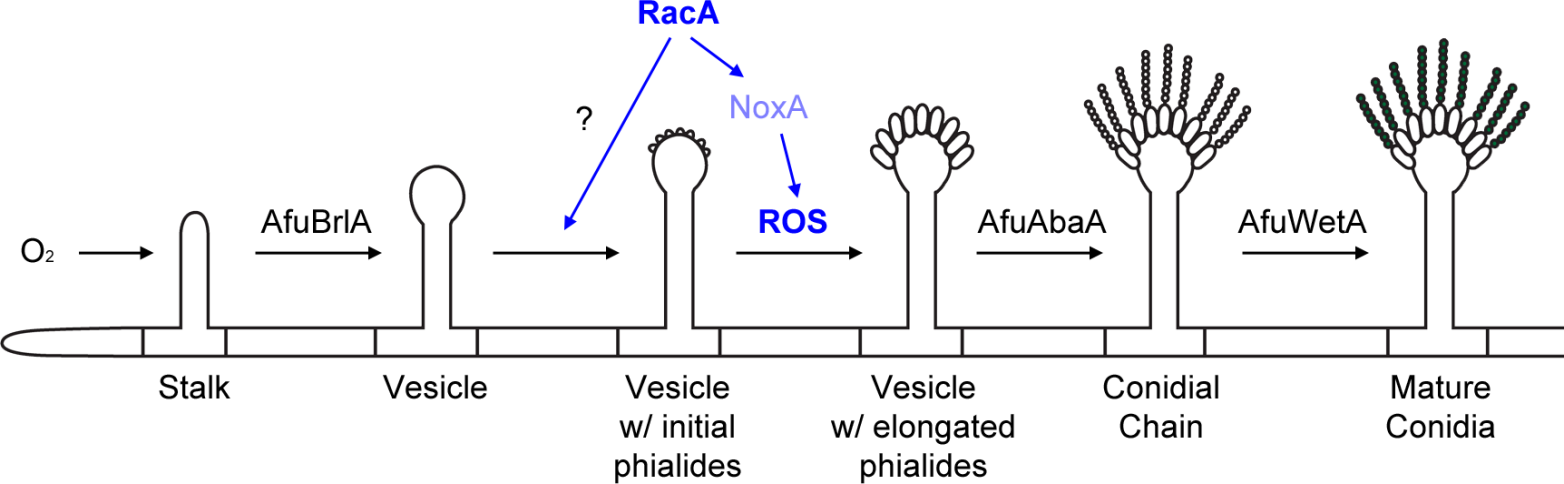
Suggested Model for conidiogenesis regulation in *A*. *fumigatus*. Current pictorial model describing the central regulatory pathway in a typical aerial environment is presented. The novel factors identified in this study are highlighted in blue.

When considered together, our results are consistent with a model in which ROS produced by the Nox complex, including the small GTPase Rac, act as a developmental signal for the polarized growth of phialide cells of the asexual conidiogenesis pathway (Fig 7). In the absence of Rac, certain environmental stimuli (eg. temperature shifting) can result in the recovery of partial ROS production; however the distribution of this ROS is irregular when compared to that of the wild type and the recovered phialides of the ***Δ****racA* mutant fail to regain their apical dominance (Fig 4). From this, we suggest that the scattered ROS generation independent of RacA is not sufficient to maintain apical dominance of *A*. *fumigatus*. This may be due to the failure of this alternative ROS to properly localize in space and/or time, or perhaps to other unknown downstream effectors of RacA.

### Relationship between oxidative signaling and the central regulatory pathway

Previously, we have shown that oxygen is one of the environmental factors inducing *brlA* expression and conidiogenesis [12], a notion which also supports these findings, as O_2_ is the substrate for Rac-mediated ROS production. The signaling role of RacA is required for phialide development between vesicles and conidia, which are regulated by BrlA and AbaA, respectively [18, 22, 29–32]. Expression analysis of these regulatory genes also showed dramatic reduction of *AfuabaA* transcription in the ***Δ****racA* mutant, while the expression level of *AfubrlA* remained higher than the constitutive β-tubulin gene. Taken together, our results lead us to propose an improved model of the central regulatory pathway of conidiogenesis, illustrated in Fig 7. Investigation of the role(s) played by other components known to be involved in fungal cell formation, including septin ring formation and actin assembly, will be required for a more thorough elucidation of multi-axes polarity development in the conidiophore vesicles of these fungi that play important and disparate roles in interactions with human society.
